# An evaluation of the Swiss staging model for hypothermia using hospital cases and case reports from the literature

**DOI:** 10.1186/s13049-019-0636-0

**Published:** 2019-06-06

**Authors:** M. Pasquier, P. N. Carron, A. Rodrigues, F. Dami, V. Frochaux, C. Sartori, T. Deslarzes, V. Rousson

**Affiliations:** 1 Emergency Department, Lausanne University Hospital, and University of Lausanne, BH 09, CHUV, 1011 Lausanne, Switzerland; 2 Medical School of the University of Lausanne, Bugnon 21, 1011 Lausanne, Switzerland; 3 Emergency Service, Hôpital du Valais, 1951 Sion, Switzerland; 4 Department of Internal Medicine, Lausanne University Hospital, BH 10, CHUV, 1011 Lausanne, Switzerland; 5 Institute of Social and Preventive Medicine, Lausanne University Hospital, route de la Corniche 10, 1010 Lausanne, Switzerland

**Keywords:** Cardiac arrest, Core temperature, Emergency medicine, Hypothermia, Swiss staging

## Abstract

**Background:**

The Swiss staging model for hypothermia uses clinical indicators to stage hypothermia and guide the management of hypothermic patients. The proposed temperature range for clinical stage 1 is < 35–32 °C, for stage 2 is < 32–28 °C, for stage 3 is < 28–24 °C, and for stage 4 is below 24 °C. Our previous study using 183 case reports from the literature showed that the measured temperature only corresponded to the clinical stage in the Swiss staging model in approximately 50% of cases. This study, however, included few patients with moderate hypothermia. We aimed to expand this database by adding cases of hypothermic patients admitted to hospital to perform a more comprehensive evaluation of the staging model.

**Methods:**

We retrospectively included patients aged ≥18 y admitted to hospital between 1.1.1994 and 15.7.2016 with a core temperature below 35 °C. We added the cases identified through our previously published literature review to estimate the percentage of those patients who were correctly classified and compare the theoretical with the observed temperature ranges for each clinical stage.

**Results:**

We included 305 cases (122 patients from the hospital sampling and the 183 previously published). Using the theoretically derived temperature ranges for clinical stages resulted in 185/305 (61%) patients being assigned to the correct temperature range. Temperature was overestimated using the clinical stage in 55/305 cases (18%) and underestimated in 65/305 cases (21%); important overlaps in temperature existed among the four stage groups. The optimal temperature thresholds for discriminating between the four stages (32.1 °C, 27.5 °C, and 24.1 °C) were close to those proposed historically (32 °C, 28 °C, and 24 °C).

**Conclusions:**

Our results provide further evidence of the relationship between the clinical state of patients and their temperature. The historical proposed temperature thresholds were almost optimal for discriminating between the different stages. Adding overlapping temperature ranges for each clinical stage might help clinicians to make appropriate decisions when using clinical signs to infer temperature. An update of the Swiss staging model for hypothermia including our methodology and findings could positively impact clinical care and future research.

## Background

The “Swiss staging classification” uses clinical information to stage the severity of hypothermia (Table 1) and was first published as part of a recommendation on the medical treatment of hypothermia published by the International Commission for Alpine Rescue [1]. This classification is widely used and integrated in the international management guidelines on accidental hypothermia [2, 3]. However, the evidence level sustaining the correlation between a proposed range of body temperatures and the clinical state of the patient is low. We previously published an evaluation based on out-of-hospital cases published in the medical literature [4]. We showed that the measured core body temperature only corresponded to the clinical stage in the Swiss staging model of hypothermia in approximately 50% of cases [4]. Globally, the core temperature of patients was lower than those proposed by the Swiss staging model according to the clinical stage of the patient [4]. One important limitation we acknowledged was the potentially important risk of publication bias [4]. Furthermore, there was a relative paucity of moderately hypothermic cases. We aimed to complete this database by adding cases of hypothermic patients admitted to hospital with the aim of performing a more comprehensive evaluation of the Swiss staging model for hypothermia.

**Table 1:** Swiss clinical staging of hypothermia

	Brown et al., 2012 (2)	Durrer et al., 2003 (1)	Typical core temperature (°C)
Stage 1	Conscious, shivering	Clear consciousness with shivering	35 to 32
Stage 2	Impaired consciousness, not shivering	Impaired consciousness without shivering	< 32 to 28
Stage 3	Unconscious, not shivering, vital signs present	Unconsciousness	< 28 to 24
Stage 4	No vital signs	Apparent death	< 24

There are minor differences between the original system developed by Durrer et al. [1] and the most recent versions [2]. Each clinical stage is associated with an estimate of core body temperature

## Methods

The design and methodology were very similar to those we previously described in the princeps study [4]. We retrospectively included patients aged ≥18 y admitted to one of the two Swiss hospital included (Lausanne and Sion, Switzerland) between 1.1.1994 and 15.7.2016 with a core temperature below 35 °C. These hospitals were selected as they share a joint ethics committee, and as their respective emergency departments regularly collaborate in research projects. We excluded patients for which data on clinical parameters and vital signs at presentation did not allow a classification using the Swiss staging model. To ensure that any impairment in consciousness could be firmly attributed to hypothermia alone, we excluded cases with any mention or suspicion of one of the following potential confounding factors: acute alcohol or other intoxication, drug overdose, hypoglycemia < 3 mmol/L, traumatic brain injury, or medical conditions that could lead to hypothermia. Cases of therapeutic and neonatal hypothermia were also excluded. A blood alcohol concentration of up to 150 mg/dL was not grounds for exclusion as this is considered the threshold at which signs of altered consciousness might occur [5]. The screening and inclusion was performed by one of the authors (AR), who accessed the patient’s medical chart. Questions were resolved by discussion with one of the other authors (MP). When in doubt, cases were not included in the database. The data collected from the included cases were entered in a spreadsheet (Excel; Microsoft, Redmond, WA, USA) that was then coded.

The following data were collected: age, sex, vital parameters, first recorded core body temperature, presence of shivering, occurrence of cardiac arrhythmia, causes of accidental hypothermia (the mechanism for hypothermia: immersion, exposure submersion, avalanche with burial of head under the snow), rewarming method, hospital survival, and neurological outcome (CPC: cerebral performance categories 1 = normal or slightly diminished cerebral function, 2 = moderate cerebral disability, 3 = severe cerebral disability, 4 = coma or vegetative state, and 5 = brain dead) [6]. Consciousness was evaluated using the Glasgow Coma Scale (GCS), the Alert-Verbal-Pain-Unresponsive (AVPU) classification, or any other descriptive clinical information available. Since we noticed in our initial study that the presence or absence of shivering was very seldom documented [4], we decided to clinically stage hypothermia solely on the basis of state of consciousness and vital signs. Information on shivering, when available was, however, collected. Cases were clinically classified as follows: stage 1 was defined as a GCS score = 15 or ‘A’ from the AVPU classification; stage 2 as a GCS score > 8 and < 15 or ‘V’ from AVPU; stage 3 as a GCS < 9 or ‘P’ or ‘U’ from AVPU; stage 4 as the absence of vital signs (respiratory rate of 0, no measurable blood pressure, no palpable pulse) and GCS = 3 or ‘U’ from AVPU [7, 8]. The study was approved by the institutional ethics committee (CER-VD 2016–01267).

### Statistical analysis

The cases identified through our previously published literature review (“literature sampling”) [4] were added to the included case of the present study (“hospital sampling”) to constitute the database. Descriptive statistics were determined for variables of interest and were expressed as frequencies, means and standard deviations, or medians and interquartile range (IQR) depending on the nature of the variables (categorical, normally distributed, quantitative but not normally distributed). Comparisons were conducted using Pearson’s Chi-square or Fischer’s exact tests, Student’s t-test, or the Wilcoxon rank-sum test accordingly. A bilateral *p*-value < 0.05 was considered to indicate a significant difference between patient groups. Three receiver operating characteristic (ROC) analyses were performed to determine the optimal temperature thresholds for discriminating between the four clinical stage groups. For every possible threshold temperature, t sensitivity was defined as the proportion of patients in the higher stage group with a temperature below t, and specificity as the proportion of patients in the lower stage group with a temperature equal to or higher than t. We used the area under the ROC curve (AUC) to summarise the discrimination between two groups. The optimal temperature to discriminate two groups was defined as the threshold t which maximises the sum of sensitivity plus specificity, which is equivalent to the Youden index [9]. Data was retrieved from the patient information database and exported into Stata version 14 (Stata Corporation, College Station, TX, USA) for analysis.

## Results

**Fig. 1:**
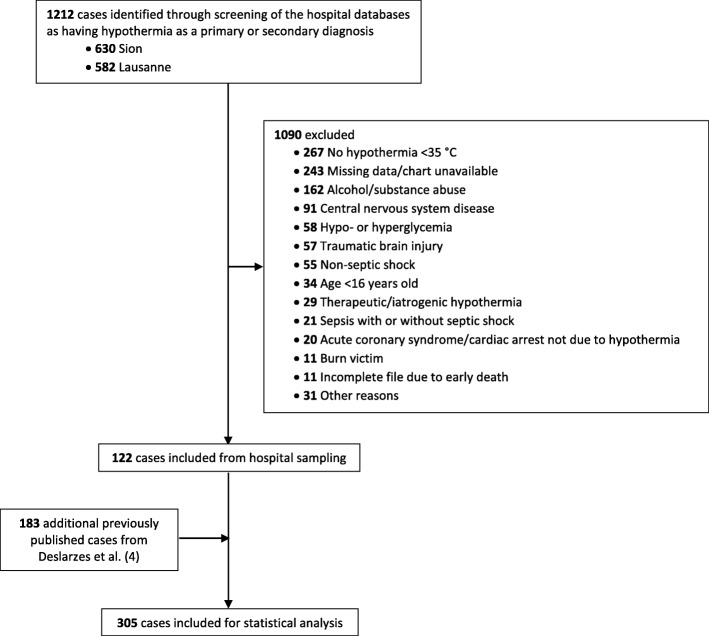
Flowchart of study cases

We included 122 patients from our hospital sampling (Fig. 1). Addition of the 183 previously published cases identified through our literature review [4] resulted in a database of 305 cases for analysis. Most (84%) of the hospital patients were in clinical stage 1 or 2, and most (81%) cases from the literature sampling were classified as stages 3 or 4 (Table 2). Shivering was mentioned only 19 times in the medical charts of hospital patients (reported as present in 14 and absent in 5 patients). Among the patients with shivering, six were in clinical stage 1, five in stage 2, and three in stage 3. Their median temperature was 30.5 °C (IQR 29–32.2; range 21–34).

**Table 2:** Comparison of baseline characteristics between the cases identified through hospital sampling and those identified from the literature review

	Overall (*n* = 305)	Hospital (*n* = 122)	Literature (*n* = 183)	*P* value
Age (years), mean ± SD	47 ± 26	56 ± 22	41 ± 27	< 0.001
Sex male, n (%)	186 (61)	75 (61)	111 (61)	0.886
Temp (°C), mean ± SD	27.7 ± 5.2	31.8 ± 3.3	25.0 ± 4.4	< 0.001
GCS, median (IQR)	7 (3–15)	15 (12–15)	3 (3–6)	< 0.001
Heart rate, mean ± SD	48 ± 39	71 ± 26	30 ± 37	< 0.001
Heart rate, median (IQR)	54 (0–80)	76 (58–90)	18 (0–54)	< 0.001
Systolic blood pressure (mmHg), mean ± SD	121 ± 31	131 ± 25	101 ± 33	< 0.001
Respiration rate, median (IQR)	0 (0–16)	18 (10–20)	0 (0–0)	< 0.001
Clinical stage, n (%)				< 0.001
1	89 (29)	79 (65)	10 (5)	
2	47 (15)	23 (19)	24 (13)	
3	79 (26)	12 (10)	68 (37)	
4	90 (30)	8 (7)	81 (44)	
Cause, n (%)				< 0.001
Water exposition	91 (30)	14 (11)	77 (42)	
Avalanche	17 (6)	9 (7)	8 (4)	
Environmental/other/unknown	197 (65)	99 (81)	98 (54)	
Survival, n (%)	262 (94)	112 (92)	150 (96)	0.195
Outcome for survivors, n (%)				0.060
CPC 1	251 (96)	111 (99)	140 (93)	
CPC 2–3	6 (2.3)	1 (0.9)	5 (3.3)	
Unknown	5 (1.9)	0 (0)	5 (3.3)	

*CPC* cerebral performance categories (1 = normal or slightly diminished cerebral function, 5 = brain dead); *GCS* Glasgow Coma Scale, *IQR* interquartile range

The correspondence between clinical stage and the measured temperature for the 305 cases is presented in Table 3. The observed mean temperature was 33.2 ± 1.6 °C for patients clinically classified as stage 1, 29.4 ± 3.0 °C for those classified as stage 2, 26.0 ± 3.4 °C for stage 3, and 22.8 ± 4.3 °C for stage 4.

**Table 3:** Correspondence between the clinical stage and the measured temperature for the 305 cases

	≥ 32 T° < 35	≥ 28 T° < 32	≥ 24 T° < 28	T° < 24	overall, N (%)	mean T ± SD^a^	range	95% CI for mean	90% prediction interval^b^
Stage 1, n (%)									
Hospital	**66**	13	0	0	79 (64.8)	33.4 ± 1.3	28.5–34.9	33.1–33.7	30.8–34.6
Literature	**4**	6	0	0	10 (5.5)	31.3 ± 2.2	28.1–34.2	29.7–32.9	28.3–34.1
Overall	**70**	19	0	0	89 (29.2)	33.2 ± 1.6	28.1–34.9	32.8–33.5	30.0–34.6
Stage 2, n (%)									
Hospital	4	**15**	3	1	23 (18.9)	30.6 ± 2.5	26.0–34.8	29.5–31.6	26.1–34.0
Literature	3	**11**	8	2	24 (13.1)	28.3 ± 3.2	22.0–34	27.0–29.6	23.3–33.9
Overall	7	**26**	11	3	47 (15.4)	29.4 ± 3.0	22.0–34.8	28.5–30.3	24.8–34.0
Stage 3, n (%)									
Hospital	1	5	**4**	2	12 (9.8)	28.1 ± 3.9	19.8–33.5	25.7–30.6	22.7–32.3
Literature	3	12	**33**	20	68 (37.2)	25.6 ± 3.2	19.3–33.4	24.9–26.4	21.0–31.6
Overall	4	17	**37**	22	80 (26.2)	26.0 ± 3.4	19.3–33.5	25.3–26.8	21.0–31.9
Stage 4, n (%)									
Hospital	0	1	2	**5**	8 (6.6)	24.5 ± 2.8	20.2–28	22.1–26.9	20.8–27.9
Literature	0	9	25	**47**	81 (44.3)	22.7 ± 4.3	13.7–31.8	21.7–23.6	15.5–28.9
Overall	0	10	27	**52**	89 (29.2)	22.8 ± 4.3	13.7–31.8	22.0–23.7	15.7–28.9

^a^ In nine cases, we retained the lowest temperature of the thermometer as the actual temperature; *p* < 0.05 for T° between hospital and literature for stages 1, 2, and 3 (but not for stage 4)

^b^ 90% prediction intervals were calculated using quantiles 5 and 95%, such that the probabilities that the temperature of an individual is found either below or above that range are both equal to 5%

*T*° = core body temperature in °C

The percentage of patients who were correctly classified by the Swiss clinical staging (i.e. whose temperature was in the range predicted by their clinical stage) was 61% (185/305, 95% CI = (55 –66%)) overall and was significantly higher (*p* < 0.001) in the hospital sampling (74%; 90/122) than the literature sampling (52%, 95/183). Overall, temperature was overestimated using the clinical stage to predict temperature in 55/305 cases (18%), and underestimated in 65/305 cases (21%).

**Fig. 2:**
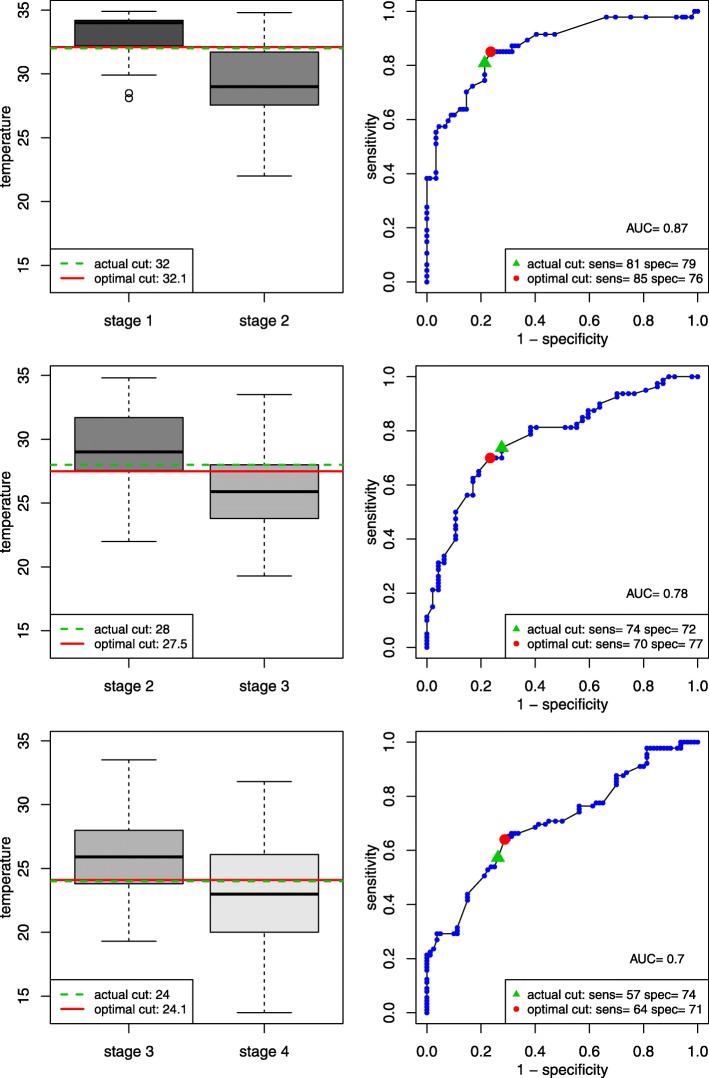
ROC curves and optimal core body temperature thresholds in °C for stage discrimination. For every possible threshold temperature, T°, sensitivity was defined as the proportion of patients in the higher stage group with a temperature below T°, and specificity as the proportion of patients in the lower stage group with a temperature equal to or above T°. The temperature that best distinguished between stages was taken as the temperature value that maximised the sum of sensitivity plus specificity. Using the estimated optimal thresholds increased (sensitivity + specificity) from 160 to 161 for the stage 1/2 threshold, from 146 to 147 for the stage 2/3 threshold, and from 131 to 135 for the Stage 3/4 threshold. Actual cut = accepted threshold for current clinical Swiss staging system; optimal cut = optimal threshold i.e. the temperature at which the sum of sensitivity + specificity is maximal; AUC = area under the curve

The results of the ROC analyses are shown in Fig. 2. The optimal temperature threshold for discriminating between stage 1 and stage 2 is 32.1 °C (actual cutoff = 32 °C), between stages 2 and 3 is 27.5 °C (actual cutoff = 28 °C), and between stages 3 and 4 is 24.1 °C (actual cutoff = 24 °C).

## Discussion

In this retrospective study of 305 cases of hypothermic patients identified through literature and hospital chart reviews, only 61% of patients had a temperature in the range predicted by their clinical stage. However, we found that the historical [1] proposed temperature thresholds (i.e. 32 °C, 28 °C, and 24 °C) were almost optimal for discriminating between the different stages. Our study findings could positively influence clinical care practices, notably regarding the on-site management of potentially hypothermic patients.

### Rate of correct classification and consequences of misclassifications

Our results provide further evidence of the performance of the Swiss staging model, in particular the rate of incorrect classifications. Using the theoretically derived temperature ranges for clinical stages would result in approximately 40% of patients being assigned to a wrong temperature range. The potential clinical consequences of such misclassifications have been extensively discussed in our first study [4]. Underestimating the patient’s temperature (which would have been the case for 21% of our patients) might lead to unnecessary patient monitoring or to incorrect hospital orientation, but probably not to any deleterious consequences apart from a waste of resources. Overestimating the temperature of the patient when using the clinical stage (which would have been the case for 18% of our patients) is more concerning. It might lead to under-treatment of the patient due to an underestimation of the risk of cardiac arrest (CA) in clinical stage 1 patients with an actual temperature < 30 °C [10], to sub-optimal patient orientation in clinical stage 2 patients with an actual temperature < 28 °C [2, 11], or to an underestimation of the magnitude of the risk of cardiac arrest in clinical stage 3 patients with an actual temperature < 24 °C [12].

### Importance of the Swiss staging model

Having a reliable measurement or estimate of a patient’s core temperature is important since some critical decisions are based on the temperature value [13, 14]. This is notably the case regarding the withholding of defibrillation after an initial three shocks in a CA patient with a temperature < 30 °C [3], the interruption in chest compressions in some selected CA patients with a temperature < 28 °C [3, 15], or the transport of non-arrested patients with a temperature < 28 °C directly to an ECLS (Extracorporeal Life Support) centre [2, 3, 11]. Temperature is also used to evaluate the risk of cardiac arrest which might occur below 30 °C [10] as well as its magnitude, which increases as temperature decreases [2, 11].

Despite the well-established and acknowledged importance of temperature to guide the management of potentially hypothermic patients, it has been shown in many different setting and countries that suitable thermometers, notably low-reading thermometers, are often lacking [13, 14, 16–20]. Obtaining an accurate temperature measurement in the prehospital setting is also subject to some limits, including in conscious patients [19, 21, 22]. The research and development of new adapted thermometers is ongoing and might provide a solution in the future [14]. However, even if it did exist, an appropriate thermometer might not always be available, and having a clinically based estimation of core temperature is thus important.

This lack of suitable tools explains why the Swiss staging model is widely used in clinical (notably prehospital) practice, as reflected by its mention in the major guidelines on hypothermia management [2, 3, 11, 23]. The Swiss staging model may be used by medical as well as non-medical care providers when an accurate core temperature measurement is not possible or available [19]. Several rescue teams are indeed not equipped with any thermometers and instead use the Swiss staging model [18]. For all these reasons, increasing the evidence level sustaining the link between the clinical state of a given patient and his/her core temperature estimation is of utmost importance.

### Practical implications of our findings and the proposal of improvements to the Swiss staging model

Our estimates of the optimal thresholds to discriminate between the different stages were remarkably close to those proposed for the original Swiss staging model (32.1 °C vs 32 °C for stage 1 and 2 groups, 27.5 °C vs. 28 °C for stage 2 and 3 groups, and 24.1 °C vs 24 °C for stage 3 and 4 groups) [1]. This study provides further evidence to support these figures. However, this study including additional data from hospital cases also confirms the important overlaps between the four stage groups with respect to core temperature [4]. This strengthens the idea that it might be preferable to associate the different stages with overlapping temperature ranges. One could typically use prediction intervals (e.g. 90%), which based on our study, would be ≥30 °C for stage 1, 25–34 °C for stage 2, 21–32 °C for stage 3, and ≤ 29 °C for stage 4.

Since it is recommended that treatment should be guided based mainly on some key clinical factors (e.g. the level of consciousness or cardiovascular stability), it is also recognised that the core temperature can provide additional helpful information [23]. It is already known that the core temperature does not always correspond to a precise level of consciousness or clinical state [19]. It has, however, been recently advocated that the correlation of clinical stage with core temperature should be better defined [12, 24]. It has also been suggested that clinicians should be aware of the limits and pitfalls of using clinical signs to infer core temperature [19]. The Swiss staging model will still be used on many occasions when accurate core temperature measurement is not possible. Our study should help clinicians to guide their management and decisions based on a more comprehensive estimation on the core temperature.

#### Limitations

Our study suffers from several limitations. First, we were unable to include the presence or absence of shivering in our analysis due to the paucity of cases for which this information was available, not among the literature cases [4], but also among the hospital charts. Shivering is mentioned in the first stage of the original Swiss staging model [1]. It has, however, been reported in patients with deep hypothermia [25, 26]. Furthermore, it might be misleading to use shivering additionally to the state of consciousness, since patients could be classified in two different stages depending on their clinical findings. We would therefore advocate analysing the relationship between shivering and temperature independently from the state of consciousness to avoid confusion. A second limitation is related to the reported body temperature of the hypothermic patients that were included in this study. We cannot be certain that effective devices were used properly. The use of an inappropriate device or a delay between measurement and the clinical evaluation of the patient could potentially have biased our results. However, we have no evidence to indicate that such a bias, if any, would be systematic. Our convenience sample of two hospitals may also be considered as a limitation. Although this may theoretically limit the external validity of our findings, we have no reason to suppose that this may induce any systematic bias. Finally, we were not able to gather reliable information on the clinical decision-making process, as well as the relevant outcomes such as the presence or occurrence or arrhythmias or hospital complications. This might have provided interesting information about the clinical consequences of potential misclassifications. Most these limitations pertain to the retrospective nature of the study. Additional prospective studies or the analysis of good-quality prospectively collected data would be needed to overcome these limitations and validate our findings.

## Conclusions

Our results provide further evidence of the relationship between the clinical state of hypothermia and the measured temperature. We suggest to define the four clinical stages using the definitions we used in this study, which would also help to guiding future research. Adding overlapping ranges of temperature for each clinical stage might help clinicians to make appropriate decisions when using clinical signs to infer core temperature. A new version of the Swiss staging model for hypothermia would be the next valuable step, which should ideally be discussed and validated by the International Commission for Alpine Rescue Alpine Emergency Medicine Commission (ICAR-MEDCOM).

## Data Availability

The patient’s data that support the findings of this study are not available without appropriate ethical approval.

## Abbreviations

AUC: Area under the curve
AVPU: Alert-Verbal-Pain-Unresponsive
CA: Cardiac arrest
CPC: Cerebral performance categories
ECLS: Extracorporeal Life Support
GCS: Glasgow Coma Scale
ICAR-MEDCOM: The International Commission for Alpine Rescue Alpine Emergency Medicine Commission
IQR: Interquartile range
ROC: Receiver operating characteristic
SD: Standard deviation
